# Detection of Somatic Mutations by High-Resolution DNA Melting (HRM) Analysis in Multiple Cancers

**DOI:** 10.1371/journal.pone.0014522

**Published:** 2011-01-17

**Authors:** Jesus Gonzalez-Bosquet, Jacob Calcei, Jun S. Wei, Montserrat Garcia-Closas, Mark E. Sherman, Stephen Hewitt, Joseph Vockley, Jolanta Lissowska, Hannah P. Yang, Javed Khan, Stephen Chanock

**Affiliations:** 1 Laboratory of Translational Genomics, Department of Health and Human Services (DHHS), National Cancer Institute (NCI), National Institutes of Health (NIH), Bethesda, Maryland, United States of America; 2 Advanced Technology Center, Pediatric Oncology Branch, Center for Cancer Research, DHHS, NCI, NIH, Bethesda, Maryland, United States of America; 3 Hormonal and Reproductive Epidemiology Branch, Division of Cancer Epidemiology and Genetics, DHHS, NCI, NIH, Bethesda, Maryland, United States of America; 4 Tissue Array Research Program, DHHS, NCI, NIH, Bethesda, Maryland, United States of America; 5 Office of Cancer Genomics, DHHS, NCI, NIH, Bethesda, Maryland, United States of America; 6 Department of Cancer Epidemiology and Prevention, M. Sklodowska-Curie Institute of Oncology and Cancer Center, Warsaw, Poland; University of Montreal, Canada

## Abstract

Identification of somatic mutations in cancer is a major goal for understanding and monitoring the events related to cancer initiation and progression. High resolution melting (HRM) curve analysis represents a fast, post-PCR high-throughput method for scanning somatic sequence alterations in target genes. The aim of this study was to assess the sensitivity and specificity of HRM analysis for tumor mutation screening in a range of tumor samples, which included 216 frozen pediatric small rounded blue-cell tumors as well as 180 paraffin-embedded tumors from breast, endometrial and ovarian cancers (60 of each). HRM analysis was performed in exons of the following candidate genes known to harbor established commonly observed mutations: *PIK3CA*, *ERBB2*, *KRAS*, *TP53*, *EGFR*, *BRAF*, *GATA3*, and *FGFR3*. Bi-directional sequencing analysis was used to determine the accuracy of the HRM analysis. For the 39 mutations observed in frozen samples, the sensitivity and specificity of HRM analysis were 97% and 87%, respectively. There were 67 mutation/variants in the paraffin-embedded samples, and the sensitivity and specificity for the HRM analysis were 88% and 80%, respectively. Paraffin-embedded samples require higher quantity of purified DNA for high performance. In summary, HRM analysis is a promising moderate-throughput screening test for mutations among known candidate genomic regions. Although the overall accuracy appears to be better in frozen specimens, somatic alterations were detected in DNA extracted from paraffin-embedded samples.

## Introduction

Recently, the first cancer genomes to be completely sequenced have revealed an unanticiapted breadth and complexity of somatic alterations [1], [2], [3]. The discovery of somatic sequence alterations, has accelerated the investigation of ir underlying mechanisms in carcinogenesis. Somatic alterations implicated in cancer development and growth advantage are called *driver* mutations. However, the majority of somatic alterations in cancer genomes are a consequence of genomci instability and appear to be *passenger* or bystander mutations that are unlikely to be involved in oncogenesis [4], [5]. Large-scale sequencing studies have shown that the prevalence and signature of somatic mutations in human cancers are highly variable [5], [6], [7], [8]. Based on these studies, we can estimate that the majority of somatic mutations in cancer cells are likely to be passenger mutations, whereas a minority are estimated to be driver mutations [5], [7]. The full landscape of the prevalence of mutations as well as their functional consequences will not be appreciated until thousands of cancer genomes have been sequenced.

Sequencing cancer genomes is a formidable task that requires expensive technologies and computational support to assemble large portions of the genome. Because of the intense interest in identifying key somatic alterations, investigation has focused on techniques for screening or analyzing regions of interest. Most studies have concentrated on coding regions and adjacent intronic or putative regulatory regions [9]. One of those techniques is the high resolution melting (HRM) curve analysis, a polymerase chain reaction (PCR) based high-throughput assay for detecting DNA sequence variation by measuring changes in the melting of a DNA duplex, that has been used successfully with DNA extracted from both frozen and paraffin-embedded tissue [10], [11], [12].

HRM specific PCR products are generated to interrogate conformational differences, also known as dissociation curve analysis, using conventional real-time PCR platforms. It is utilized in combination with a double stranded DNA binding dye in order to characterize primer-related non-specific amplification (or primer dimer) for detection of a specific target. Single-base changes in PCR products are detected by altered HRM properties monitored through the release of fluorescent double strand DNA binding dye [13], [14]. The development of accurate and inexpensive instruments that offer HRM capabilities, and new fluorescent dyes, make this method attractive for targeted mutation scanning and also germ line genotyping. HRM analysis is utilized to pre-scan candidate genes suspicious of harboring mutations, reducing significantly the amount of DNA sequencing to be performed [15], [16], [17], [18], [19], [20].

The aim of this study was to assess the sensitivity and specificity of an inexpensive HRM analysis platform for mutation scanning of single-base variation in a range of tumor samples: frozen pediatric small rounded blue-cell tumors and paraffin-embedded tumors from breast, endometrium and ovarian cancers. Bi-directional sequence analysis was performed to determine the accuracy of this DNA HRM technology.

## Methods

### Ethics Statement

The Institutional Review Board for the Polish Breast, Ovarian, and Endometrial Cancer Study were approved by the National Cancer Institute (NCI), at Bethesda, MD, the M. Sklodowska Institute of Oncology and Cancer Center in Warsaw, and the Institute of Occupational Medicine (IOM) in Lodz, both in Poland [21]. Written informed consent for participation on the studies was obtained at the participating institutions from all participants involved.

All frozen samples from pediatric small rounded blue-cell tumors and obtained from Cooperative Human Tissue Network (http://chtn.nci.nih.gov/), were anonymized, and our protocol was reviewed by the Office of Human Subjects Research at National Institutes of Health, Bethesda, MD, and deemed exempt.

### DNA samples

#### Frozen tissue samples

Snap frozen tumor samples were obtained from Cooperative Human Tissue Network (http://chtn.nci.nih.gov/). Neuroblastoma cell lines and their culture conditions are described elsewhere [22]. Genomic DNA was extracted from frozen primary tumor samples (neuroblastoma, n = 140; rhabdomyosarcoma, n = 63) and neuroblastoma cell lines (n = 13) using a published protocol [23]. DNA concentration was quantified using NanoDrop (Thermo Fisher Scientific, Wilmington, DE), and then adjusted to the same concentration, 10 ng/µL, for the 12 assays. Matched control genomic DNA was available from peripheral blood for 43 cancers.

#### Paraffin-embedded tissue samples

The Polish Breast, Ovarian, and Endometrial Cancer Study is part of a collaborative study between the U.S. National Cancer Institute (NCI), the M. Sklodowska Institute of Oncology and Cancer Center in Warsaw, and the Institute of Occupational Medicine (IOM) in Lodz [21] designed to study risk factors for breast, endometrial and ovarian cancer [24], [25], [26]. Paraffin blocks of tumor tissue from participants of this study that underwent surgery were collected. In total, we included tissue from 60 participants with breast cancer, 60 with endometrial cancer and 60 with ovarian cancer.

Single 0.6 mm tissue cores targeted to areas with tumor that had been identified and marked by a pathologist (MES) were obtained from each tumor block for DNA extraction, using a tissue microarray coring needle for each sample. Microdissection was performed for a small proportion of the samples, making it difficult to accurately assess the percentage of tumor material. Nucleic acid extraction was performed with the Agencourt® FormaPure™ kit (Agencourt Bioscience Corporation, Beverly, MA) according to the manufacturer's instructions. To avoid interferences with the PCR we removed RNA from purified total nucleic acid during the extraction method. After extraction and purification, DNA concentration and purity were quantified using NanoDrop (Thermo Fisher Scientific, Wilmington, DE). Total genomic DNA extracted with this method yielded an averaged of 2.07 µg (range 0.03 to 7.69 µg). The purity of DNA for each extraction method was assessed by measuring the intensity of absorbance of the DNA solution at wavelengths 260 nm (A260) and 280 nm (A280).

### Selection of exons for screening

The set of exons selected for this mutation scanning analysis were drawn from cancer genes frequently mutated (*PIK3CA*, *ERBB2*, *KRAS*, *TP53*, *EGFR*, *BRAF*, *GATA3*, and *FGFR3*) in published reports, with a particular emphasis on breast, ovarian and endometrium cancers [5], [9], [27], [28], [29], [30], [31], [32]. Also, HRM analysis for these particular genomic regions had already been optimized.

### Primers and pre-HRM PCR

Primers of the exons, as well as the size of the amplicons, used for the pre-HRM PCR are listed in Table 1. On average, 40 bp of the proximal - or 5′- intronic region flanking the target exon and 41 bp of the 3′ intronic region flanking the target exon were covered by the amplicon. The only exception was exon 6 of *GATA3*, which measures 1462 bp, of which 284 bp correspond to coding nucleotides. In this particular case, the amplicon did not extend over the 3′ side of the intron (Table 1 for details).

**Table 1:** Primers for pre-HRM DNA amplification.

Gene	Exon	Size (bp)	Direction primer	Primer	Intron 5′ coverage (bp)	Intron 3′ coverage (bp)
*PIK3CA*	10	258	Forward	ATCCAGAGGGGAAAAATATGAC	58	-
			Reverse	TGAGATCAGCCAAATTCAGTTAT	-	74
*FGFR3*	13	195	Forward	TGCCTCCCACCCCTTCC	21	-
			Reverse	AGGCGTCCTACTGGCAT	-	51
*ERBB2*	25	200	Forward	ACATGGGTGCTTCCCATTC	22	-
			Reverse	GCTCCTTGGTCCTTCACCTA	-	22
*TP53*	5^1^	186	Forward	GCCCTGACTTTCAACTCTG	39	-
			Reverse	CCTCACAACCTCCGTCAT	-	-
*TP53*	5^2^	115	Forward	TGGCCATCTACAAGCAGTC	-	-
			Reverse	CAGCCCTGTCGTCTCTC	-	34
*TP53*	7	200	Forward	GGCGCACTGGCCTCATCT	39	-
			Reverse	AGAGGCTGGGGCACAGCA	-	51
*EGFR*	23	213	Forward	CAGCAGGGTCTTCTCTGTTTC	23	-
			Reverse	GAAAATGCTGGCTGACCTAAAG	-	34
*KRAS*	2	208	Forward	GTGACATGTTCTAATATAGTCACATTTTC	46	-
			Reverse	GGTCCTGCACCAGTAATATG	-	40
*BRAF*	15	184	Forward	AGATCTACTGTTTTCCTTTACTTACTACACC	35	-
			Reverse	AATCAGTGGAAAAATAGCCTCAATTCT	-	30
*GATA3*	5	190	Forward	GATTTCACCCTCTCCTCTCTCCC	32	-
			Reverse	AGCCCTGTTCTTGCTGATCC	-	32
*GATA3*	6^1^	194	Forward	GTGGAACCCTTCTTGGTGTG	88	-
			Reverse	AGTCCTCCAGTGAGTCATGC	-	-
*GATA3*	6^2^	154	Forward	AAATGTCTAGCAAATCCAAAAAGTGCAA	-	-
			Reverse	GTGGTCAGCATGTGGCTGGA	-	-79

1 Proximal region.

2 Distal region.

Attention to detail in pre-HRM PCR conditions is paramount for optimization: 1) design of PCR primers to keep GC content under or as close as 60% as possible, product size around 200 bp and avoid known variants within the primer region; 2) selection of optimal annealing temperatures with gradient PCR; 3) and design of PCR experiments in a consistent manner: same assay, with same sample batch and same machine run.

PCR-based analyses for the different genes were performed in 96-well format with 10 µL volumes and included 10 ng of genomic DNA for frozen samples, and 1 µL of solution containing genomic DNA for paraffin-embedded tissue samples, with mean concentration of 25.8 ng/µL (SD = 21.7), and ranging from 2 to over 55 ng/µL (first quartile 8.4 ng/µL, and third quartile 36.3 ng/µL). Master Mix that included all deoxynucleoside triphosphates, Taq polymerase, and the LCGreen® PLUS (Idaho Technology, Salt Lake City, UT) was used for the pre-HRM PCR. PCR was performed using a MJ Research PTC 225 Thermal Cycler (MJ Research, GMI Inc., Ramsey, MN) with an initial denaturation at 95°C for 2 minutes, followed by 45 cycles of 2 step temperature cycling of 95°C for 30 seconds, and 66 to 70°C for 30 seconds (*PIK3CA*, *ERBB2*, *KRAS* at 66°C; *TP53*, *EGFR*, *BRAF* at 68°C; *GATA3*, *FGFR3* at 70°C). After PCR, the samples were heated to 93°C for 30 seconds and then cooled to 25°C before HRM.

### Sample handling

**Frozen.** HRM analyses were performed in duplicate on all the samples yielding either frank (n = 59) or minimal variations (n = 99) in the normalized HRM curve, and also in 20% randomly chosen negative samples (n = 45). A second round of HRM was performed to assess the reproducibility of the method, using known negative controls and positive controls. Frank variations were defined as those HRM curves interpreted by the software to be suspicious of harboring a nucleotide change or a mutation/variant, and were represented in red in the graphics (Figure S1). Minimal variation on a sample was considered when the software detected minor variance in the HRM curve with respect to the averaged wild-type curve without calling it a mutation (3% of all calls) (Figure S1). These samples were represented either in grey or green, depending on their degree of separation with the averaged wild-type curve. All samples with frank or minimal variation of the curve underwent a repeated HRM analysis, and were also sequenced.

**Paraffin-embedded.** We analyzed 60 breast cancer samples, 60 endometrial cancer samples and 60 ovarian cancer samples. The quality of the extracted DNA was measured by the presence of pre-HRM PCR product in the HRM analysis and by the presence of a single band on a 1.5% agarose gel [33]. In this set, all samples were bi-directionally sequenced.

### HRM Curve Analysis

Samples were amplified in 96-well plates, and HRM curves acquisition was performed on a prototype version of the HRM instrument, LightScanner™, using LCGreen® Plus+ Dye (Idaho Technology, Salt Lake City, UT). Depending on the assay combination on the plate, HRM range was set to accommodate each assay individual profile with at least 4°C prior to the first melt transition on the plate, with a slope of 0.3°C/s, and at least 3 degrees after the last fragment has completely melted.

Since HRM was performed in this study as the screening technology, the curves were analyzed using custom LightScanner™ software (Idaho Technology, Salt Lake City, UT). Normalization and background subtraction were first performed by fitting an exponential to the background surrounding the HRM transitions of interest. The normalized HRM curves were temperature-overlaid, to eliminate slight temperature errors between wells or runs. Difference plots of these normalized and temperature-overlaid curves were obtained by taking the fluorescence difference of each curve from the average wild-type curve at all temperature points [13], [14]. HRM curves with a plot interpreted by the software to be different from the averaged wild-type curve were considered to be suspicious of harboring a nucleotide change or a mutation/variant (Figure S1). These analytical methods have been applied previously to mutation scanning [34], [35].

### Bi-directional Sequence Analysis

Bi-directional sequence analysis was performed with primers that were designed by extending each oligonucleotide used in the pre-HRM PCR with a universal sequencing primer: M13 forward (TGTAAAACGACGGCCAGT) or M13 reverse (CAGGAAACAGCTATGACC). PCR conditions for sequencing analysis were performed in 96-well format with 10 µL volumes and included 1 µL of amplified DNA from the pre-HRM PCR reaction. Genomic DNA was used only when the sequence reaction failed with amplified DNA. PCR products were sequenced using a modified ABI Prism® BigDye Terminator protocol (Applied Biosystems, Foster City, CA). Unincorporated dyes terminators and salts were removed utilizing a Sephadex G-50 (Sigma, St Louis, MO) spin columns in a MultiScreen®-HV 96-well filter plate (Millipore, Billerica, MA). The reactions were run on an ABI 3730XL (Applied Biosystems, Foster City, CA). Sequence traces were analyzed and compared using two software packages (SeqScape™ v2.5 and Variant Reporter™ v1.0, both from Applied Biosystems, Foster City, CA) and reviewed by two independent reviewers [9]. When the software was unable to align and assemble the forward and the reverse sequences the sample was considered to have failed the sequencing process for the purpose of this study. For the paraffin-embedded assays that did not performed as well as their frozen counterparts (specifically exons from genes *TP53* and *GATA3*), PCR conditions as well as their primers were modified to improve sequencing. This included generating primers that extended across more of the genomic regions or slide 20–30 bp up or down stream. But we noted that the new, specific assays failed to optimize while testing different regions would alter the purpose of the study. In summary, sequencing error rate was 2.5% for frozen specimens and 20.0% for paraffin-embedded.

Nucleotide changes detected by sequencing were classified as novel alterations or known SNPs (or Single Nucleotide Polymorphisms) if found in dbSNP, Build 130, from the NCBI (www.ncbi.nlm.nih.gov/projects/SNP/), using Genewindow (genewindow.nci.nih.gov) [36].

### Statistical Analysis

The association between the quantity of DNA extracted from the paraffin-embedded tissue (levels: ≤10 ng/µL, 11–20 ng/µL, 21–30 ng/µL, and >30 ng/µL) and a successful pre-HRM PCR assay, measured either by the presence of a single amplicon in agarose gel or the presence of a DNA product at the HRM analysis, was performed by logistic regression analysis. Agreement between 2 variables (or reliability) was determined by a Kappa test. Kappa values less than 0.40 indicate low association, between 0.40 and 0.75 indicate medium association, and values greater than 0.75 indicate high association between two measures. Screening capabilities of HRM and the consistency of the analysis were measure using classical metrics, such as sensitivity, specificity, false negative and positive rates, considering sequencing analysis as the standard measurement for both frozen and paraffin-embedded extracted samples.

Statistical analyses were conducted using Microsoft® Excel (Redmond, WA) and R statistical package (www.r-project.org/).

## Results

### Frozen samples

Mutation screening was performed on 12 amplicons for each of 216 frozen samples during the initial HRM analysis (2,592 different determinations). We observed 59 HRM positive samples, 2510 HRM negative, and only 23 of these measurements were not evaluable (less than 1%). For the repeat HRM experiments, 47 were positive, 156 negative, and only 1 out of 204 was not evaluable. In total, 2,772 out of 2,796 (2,592 in first HRM round, and 204 in the second HRM round), or 99.1%, experiments were evaluable for screening of mutations/variants by HRM analysis.

In the initial round of analysis, 4 assays had no mutation detected: *ERBB2* exon 25, the distal region of *TP53* exon 5, *GATA3* exon 5, and the proximal region of *GATA3* exon 6. For the remainder tested exons, between 1 to 9 putative nucleotide substitutions were detected; notably exon 13 of *FGFR3* had the highest number, 30. The results of mutation screening by HRM technology in both experiments, initial and repeat, as well as the validation with sequencing for frozen samples are displayed in Table 2.

**Table 2:** Results of mutation screening by HRM (initial screen and repeat) and sequencing from frozen samples.

	HRM positive	HRM negative	Sequencing positive	Sequencing negative	Repeated HRM positive	Repeated HRM negative
*PIK3CA*×10	2	213	2	5	2	5
*ERBB2*×25	0	215	0	8	0	8
*KRAS*×2	3	213	5	5	3	5
*TP53*×5(1)	5	206	3	7	5	7
*TP53*×5(2)	0	215	0	15	1	14
*TP53*×7	9	207	5	15	7	13
*EGFR*×23	6	210	6	8	6	8
*BRAF*×15	3	213	1	13	2	12
*GATA3*×5	0	216	0	8	0	8
*GATA3*×6(1)	0	216	0	10	0	10
*GATA3*×6(2)	1	215	1	16	1	16
*FGFR3*×13	30	171	16	54	20	50
Total	59	2,510	39	164	47	156

HRM experiments were repeated on all the samples with evidence of a putative mutation on the HRM curve, and also in a subset of negative samples. The agreement between the initial and repeat screen of HRM experiments was 91%, with a kappa test value of 0.77, or high concordance between both. In general, HRM curves presented similar shapes in both independent analyses (Figure S2). The majority of disagreements resided in samples called abnormal in the initial screen and normal, or without mutation, in the repeat HRM experiment (n = 12), that were confirmed normal by sequencing. Only 1 repeated HRM analysis failed to detect a nucleotide substitution in a sample with respect to the initial screen. Later sequencing of this sample detected a substitution of reference GG alleles, at position 7518234 of chromosome 17 (exon 7 of gene *TP53*), by AA.

The sensitivity and specificity for mutation/variant screening were 97% and 87% respectively when compared to bi-directional sequencing, with a false negative rate of 3%. The overall accuracy of the test was 89% (Table 3). When the second, repeated HRM analysis was compared to sequencing results, specificity increased to 94%, as well as the accuracy, 94% (kappa of 0.82); but the false negative rate also increased to 5%. Details of sequencing results for mutations are described in Table 4. One false negative was detected when comparing HRM experiments with sequencing, failing to detect a nucleotide change, from AA to AG, in both the initial and repeat screens. This variant turned out to be a known SNP in exon 13 of *FGFR3*, rs7688609 (Table 4). Notably, comprehensive public databases (dbSNP and Ensembl) indicated G as the ancestral, reference allele in the DNA sequence for this locus, but the majority of sequenced samples in our study, 63 out of 67 (or 94%), were homozygous for AA, and only 6% were heterozygous for G (GA). Given this disparity on allele frequencies with available public data, we decided to sequence all 3 populations of HapMap as well as the SNP500 population for this particular amplicon (n = 366) [37], [38]. Overall, allele A frequency was 94%, and allele G frequency was 6%. Yoruba population presented the highest G frequency with 17%. At the same time, we sequenced this region for 43 available germ line DNA from the patients suffering from these pediatric tumors; all of them were homozygous for AA. After sequencing all paraffin-embedded samples, we found similar allele frequencies.

**Table 3:** Comparison between HRM mutation screening (initial screen) and sequencing of frozen samples.

	+ Sequencing	− Sequencing	Total
+ HRM	38	21	59
− HRM	1	143	144
Total	39	164	203

Sensibility: 0.97.

Specificity: 0.87.

False positive: 0.13.

False negative: 0.03.

Accuracy: 0.89.

Note: These calculations are based uniquely on samples successfully sequenced.

**Table 4:** Mutation details from the sequencing analysis of frozen samples.

Gene	Exon	Chromosome	Location	Nucleotide	Known SNP	Samples affected
*PIK3CA*	10	3	180418785	G/A	No	1
	10	3	180418867	A/T	No	1
*FGFR3* **	13	4	1777618	G/A	No	1
	13	4	1777626	C/T	No	1
	13	4	1777647	C/G	No	1
	13	4	1777674	C/T	No	1
	13	4	1777692	A/G**	rs7688609	4
	13	4	1777713	G/C	rs17886888	1
	13	4	1777720	G/A	rs3135898	9
*ERBB2*	25	17	-		-	-
*TP53*	5(1)	17	7519251	G/T	No	2
	5(1)	17	7519188	G/A	No	1
	5(1)	17	7519200	Del(cgcccggcaccc)	No	1
	5(1)	17	7519198	Del(cccggcaccc)	No	1
	5(2)	17	-		-	-
*TP53* *	7	17	7518317	C/T*	No	1
	7	17	7518315	A/C*	No	1
	7	17	7518269	T/G	TP53-147 (Poly-0023190)	1
	7	17	7518264	C/T	TP53-148 (Poly-0023191)	1
	7	17	7518263	G/A	rs11540652	1
	7	17	7518234	G/A	No	1
*EGFR*	23	7	55226944	C/T	rs17290559	6
*KRAS*	2	12	25289548	G/A	No	2
	2	12	25289551	G/A	No	1
*BRAF*	15	7	140099605	T/A	BRAF-01(Poly-0019246)	1
*GATA3*	5	10	-		-	-
*GATA3*	6(1)	10	-		-	-
	6(2)	10	8155836	C/T	GATA3-54 (Poly-0008004)	1

*One sample had 2 mutation/variants.

**Both, dbSNP and Ensembl, appoint G as the ancestral allele; but the overall allele frequency in both reports was 96% for A and 4% for G.

### Paraffin-embedded samples

The A260/A280 ratio, a measure of the purity of the paraffin-embedded extracted DNA, had a mean of 1.92 (SD = 0.45) for all breast cancer samples, a mean of 1.82 (SD = 0.12) for endometrial cancer samples, and a mean of 2.0 (SD = 0.27) for ovarian cancer samples. There was a direct association between the concentration of DNA (in ng/µL) extracted from the paraffin-embedded samples, the DNA amount used for the pre-HRM PCR, and the presence of a single band in the agarose gel (p<0.001). Also there was an association between extracted DNA concentration and the presence of an adequate HRM curve for analysis (p<0.001). HRM was successful 96% of the time when the quantity of paraffin-embedded extracted DNA used for this technique was more than 30 ng in comparison with 92% when the quantity was ≤30 ng (Table 5).

**Table 5:** Correlation between the quantity of DNA extracted from the paraffin-embedded tissue, used for pre-HRM PCR, and a band in the gel (p<0.001). Also, correlation between DNA quantity and the presence of a HRM curve (p<0.001).

	Band in gel			
DNA (ng/µL)	Yes	No	Total	%
0–30	444	72	516	0.86
>30	196	8	204	0.96
Total	640	80	720	0.89

	HRM curve			
DNA (ng/µL)	Yes	No	Total	%
0–30	1,272	114	1,386	0.92
>30	736	31	767	0.96
Total	2,008	145	2,153	0.93

Overall, 93% (2,008 out of 2,153) of the measurements of paraffin-embedded samples by HRM analysis were evaluable. This technique was more successful when frozen DNA specimens were analyzed than when DNA extracted from paraffin-embedded samples was used, 99.1% versus 93.3% (p<0.001).

The results of the mutation screening by HRM technology and its validation with bi-directional sequence analysis for the paraffin-embedded samples are displayed in Table 6. Also, a representation of these results is portrayed in Figure S3. The overall sensitivity and specificity for the samples suspicious for mutation in the HRM analysis were 88% and 80% respectively when compared to sequencing, with a false negative rate of 12% (Table 7). However, a relatively small percentage of DNA extracted from paraffin-embedded samples was difficult to sequence. As we mentioned, the sequence reaction was undertaken using amplified DNA from the pre-HRM PCR. When sequencing of a particular assay failed, genomic DNA was utilized and sequencing repeated. With this strategy, confirmatory sequence in frozen samples could be performed over 97% of the time. This was not the case with DNA extracted from paraffin-embedded tissue, where sequencing was achieved 80% of the time using the same strategy. In particular, the success rate of DNA sequencing from paraffin-embedded tissue was less than 50% for exons from genes *TP53* (distal region of exon 5, and exon 7) and *GATA3* (proximal region of exon 6), affecting the comparison between sequence analysis and HRM curves. Sequencing of these amplicons failed in 341 out of 397 reactions, which accounted for 86% of all failed sequencing. When these failed assays were excluded from the comparison between HRM curves and sequencing, sensitivity increased to 92%, with an accuracy of 80%. Paraffin-embedded sequencing details are described in Table S1. All new nucleotides changes found on the study samples were submitted to dbSNP (build 131) and displayed in Table S2.

**Table 6:** Results of mutation screening by HRM and sequencing from the paraffin-embedded samples.

	HRM +	HRM −	Total	Sequencing +	Sequencing −
*PIK3CA*×10	26	73	**99**	6	93
*ERBB2*×25	44	130	**174**	7	167
*KRAS*×2	75	98	**173**	24	149
*TP53*×5–1	13	158	**171**	7	164
*TP53*×5–2	20	76	**96**	5	91
*TP53*×7	3	27	**30**	2	28
*EGFR*×23	26	146	**172**	7	165
*BRAF*×15	26	142	**168**	1	167
*GATA*×5	34	127	**161**	3	158
*GATA*×6–1	8	50	**58**	0	58
*GATA*×6–2	36	124	**160**	0	160
*FGFR3*×13	53	71	**124**	5	119
Total	364	1,222	**1,586**	67	1,519

**Table 7:** Comparison between HRM mutation screening and sequencing of paraffin-embedded samples.

	+ Sequencing	− Sequencing	Total
+ HRM	59	305	364
− HRM	8	1,214	1,222
Total	67	1,519	1,586

Sensibility: 0.88.

Specificity: 0.80.

False positive: 0.20.

False negative: 0.12.

Accuracy: 0.80.

Note: These calculations are based uniquely on samples successfully sequenced.

## Discussion

HRM analysis using the LightScanner™ represents a moderate-throughput screening test for mutations among candidate genomic regions. The comparison with bi-directional sequencing analysis provides strong evidence for its accuracy despite the low prevalence mutation/variant rate, particularly since selected exons harbored no mutations. Our results are consistent with earlier reports [28], [39]. The observed rate was 39 for 2,569 (or 1.5%) mutation/variant for all analyzed exons within the 216 frozen pediatric small rounded blue-cell tumors, and 67 for 1,586 (or 4.2%) mutation/variant for all analyzed exons in the 180 gynecological solid tumors (Tables 4 and S1, respectively).

Sensitivity and specificity of HRM analysis was higher in frozen samples compared to paraffin-embedded samples, with an observed sensitivity of 97% for DNA extracted from frozen samples whereas it is 88% for DNA extracted from paraffin-embedded tissue. Lower performance of some assays when comparing DNA from paraffin-embedded specimens versus frozen samples has also been described in previous studies for *KRAS* and *EGFR* [12]. These differences are due, at least to some extent, to sequence alterations in DNA related to cross-linking between proteins and DNA, and inversely correlated with the number of cells within the samples [40], [41], [42]. Also, the presence of multiple mutations and point deletions may alter the efficiency of the assay, possibly the reason for low performance in *TP53* assays [16], [43]. The majority of HRM studies performed to date have concluded that, with some limitations, this is a relatively simple, rapid and inexpensive technique for detecting genomic variation in paraffin-embedded tissue samples [43]; with consistent reports on some of the genes screened on our study [11], [16], [40], [44]. Its limitations are related to a lower efficiency on regions with deletions (or insertions), on detecting homozygous variations (when compared to heterozigous), on specific assays, and the lack of agreement on the optimal length of PCR product or melting domains per amplicon [12], [13], [16].

In order to eliminate the subtle differences in the reagent components between the final elution buffers from multiple extraction platforms and to minimize variability within samples our approach was to perform the DNA extraction using a common extraction platform, conditions and protocol. With optimized sample handling and standardized DNA extraction, it is possible to screen paraffin-embedded samples with higher sensitivity [40], [41]. Despite the increased fragmentation of DNA extracted from paraffin-embedded tissue, it is possible to reliably screen shorter amplification products up to 250 bp in length. In addition, the extent of DNA fragmentation correlates with tissue type [12], [45], [46]. Success on both, HRM curve and sequencing analyses, is over 97% when 10 ng of DNA is used from frozen samples. But those results could not be achieved with the same quantity of paraffin-embedded extracted DNA (successful HRM analysis in 84%). By increasing the quantity of purified DNA added to the pre-HRM PCR ≤30 ng performance improved, partially overcoming the challenge posed by sub-optimal double stranded DNA. Optimization of pre-HRM PCR can also mitigate reduced sensitivity, especially while using special dye chemistry [46].

DNA extracted from paraffin-embedded tissues was also more difficult to sequence than DNA from frozen tissue [47], [48]. However, the objective of this study was not to establish an optimized protocol for sequencing of DNA extracted from paraffin-embedded tissue, but to assess the screening capabilities of the HRM analysis. Once optimal experimental conditions for HRM and sequencing analyses on the frozen samples were determined, we applied them to the paraffin-embedded set, to attain a fair comparison between both sets of samples. Protocol modifications of sequencing experiments could modestly improve performance, such as the use of whole genome amplification [49], [50], but this can introduce loss of heterozygosity. Steps to optimize sequencing can also include alternative primers or denaturation conditions.

Based on these considerations, our recommendations to maintain and, perhaps, enhance the screening capabilities of HRM analysis for paraffin-embedded extracted samples with a LightScanner™ would include the following:

- Inclusion of ≥30 ng total genomic DNA may increase HRM analysis success rate up to 96%.
- Pre-HRM PCR optimization should include careful primer selection to reduce GC content, adequate amplicon size, and optimal annealing temperature.
- Amplicons that failed sequencing over 50% of the times also performed poorly during HRM analysis. So it may be worthwhile to test the selected amplicons by sequencing a few samples at the beginning of the experiment.

The false positive rate of HRM analysis in paraffin embedded samples was approximately 20%, which implies that additional sequencing is needed to improve accuracy in the subset of samples with a putative mutation [12]. HRM analysis on frozen samples only considered 59 of them abnormal, for 39 with real mutation/variants. Thus, it would be necessary to sequence fewer samples for each mutation. Therefore, not only the performance is better in frozen samples with respect to paraffin-embedded samples, but also cost-efficiency.

In conclusion, HRM analysis with the LightScanner™ is a promising screening tool for mutation/variant in somatic DNA extracted from either frozen or paraffin-embedded samples, although overall accuracy is better in frozen specimens, probably related to DNA quality. This method is able to detect mutations as well as known SNPs, even in genomic regions with a low mutation prevalence rate in the range of 5% or perhaps lower.

## Supporting Information

Figure S1 Representation of HRM curve of *BRAF* exon 15 from genomic DNA extracted from frozen samples. Red arrow: HRM curves with a plot interpreted by the software to be suspicious of harboring a nucleotide change or a mutation/variant. HRM was repeated for all these samples, and all of them were sequenced. Green arrows: HRM curves with minimal variations with respect to the averaged wild-type curve. All these samples also were sequenced and HRM was repeated. Black arrow: All normalized HRM curves considered to have a wild-type sequence. 20% of these samples were randomly chosen to be repeated and sequenced as negative controls.
(2.49 MB TIF)

Figure S2 Example of HRM output from genomic DNA from tumor frozen samples set. A. Output of one of the 3 plates used for the initial analysis of exon 2 of *KRAS*. Each square represents a well: brown squares are negative controls; grey squares represent samples with no mutation/variant; red squares represent possible mutation/variant; and green are unknown for mutation/variant. B. Normalized HRM curve from the same samples in the exon 2 of *KRAS* initial analysis. C. Normalized HRM curve of the repeated KRAS analysis.
(0.68 MB TIF)

Figure S3 Samples of mutation screening with HRM technology and its validation with sequencing from paraffin-embedded samples. A. One of the assays (*EGFR*) performed in paraffin-embedded samples from breast cancer: 1. Normalized HRM curves of the assay; 2. Segment of sample assembled trace after sequencing, with the presence of a variant, where AA has replaced both alleles GG. B. Endometrial paraffin-embedded samples for *KRAS*. 1. Normalized HRM curves of the assay with elevated number of positives samples observed in the HRM curves from paraffin specimens compare to frozen samples; 2. Genotype GG has been substituted by GA.
(9.83 MB TIF)

Table S1 Mutation details from the sequencing analysis of paraffin-embedded samples.
(0.09 MB DOC)

Table S2 New nucleotides changes found on the study samples submitted to dbSNP (build 131).
(0.09 MB DOC)
