# Supplementary figures and images for: Detection of Somatic Mutations by High-Resolution DNA Melting (HRM) Analysis in Multiple Cancers
Source: PLoS One. 2011 Jan 17;6(1):e14522. doi: 10.1371/journal.pone.0014522 (PMC3022009; doi:10.1371/journal.pone.0014522)

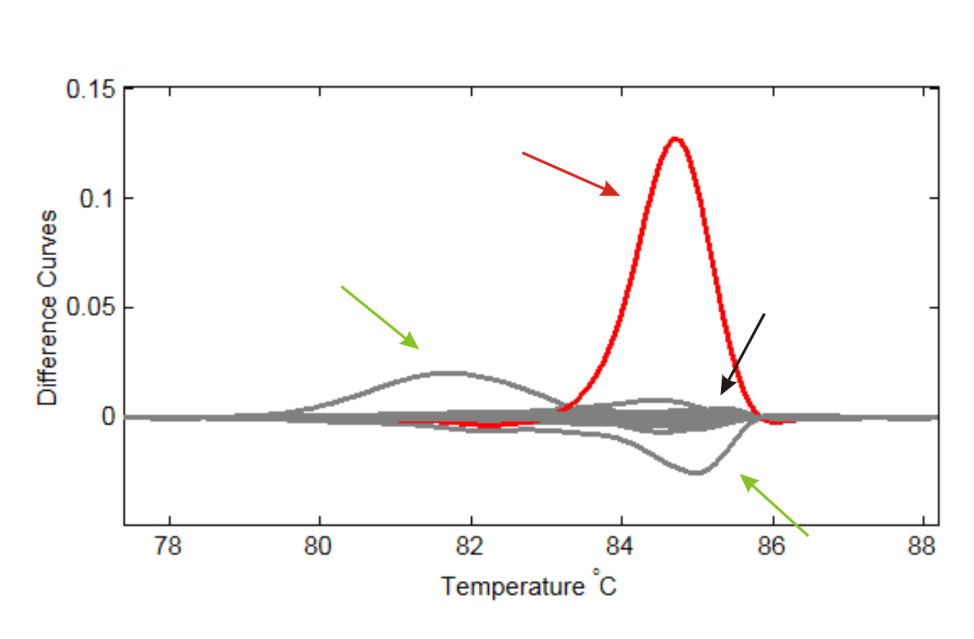

Supplement: Figure S1 — Representation of HRM curve of BRAF exon 15 from genomic DNA extracted from frozen samples. Red arrow: HRM curves with a plot interpreted by the software to be suspicious of harboring a nucleotide change or a mutation/variant. HRM was repeated for all these samples, and all of them were sequenced. Green arrows: HRM curves with minimal variations with respect to the averaged wild-type curve. All these samples also were sequenced and HRM was repeated. Black arrow: All normalized HRM curves considered to have a wild-type sequence. 20% of these samples were randomly chosen to be repeated and sequenced as negative controls. (2.49 MB TIF) [file pone.0014522.s001.tif]

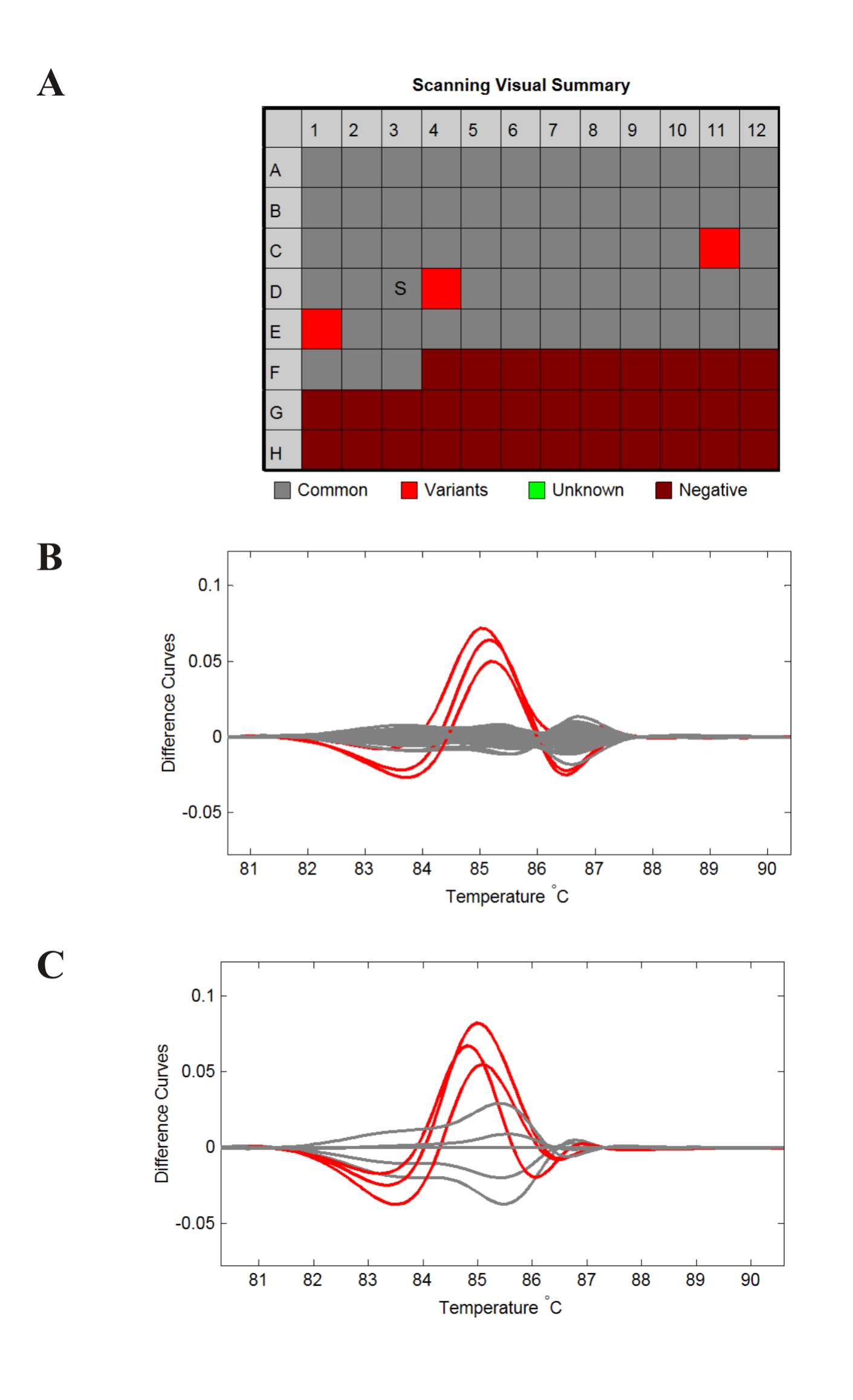

Supplement: Figure S2 — Example of HRM output from genomic DNA from tumor frozen samples set. A. Output of one of the 3 plates used for the initial analysis of exon 2 of KRAS. Each square represents a well: brown squares are negative controls; grey squares represent samples with no mutation/variant; red squares represent possible mutation/variant; and green are unknown for mutation/variant. B. Normalized HRM curve from the same samples in the exon 2 of KRAS initial analysis. C. Normalized HRM curve of the repeated KRAS analysis. (0.68 MB TIF) [file pone.0014522.s002.tif]

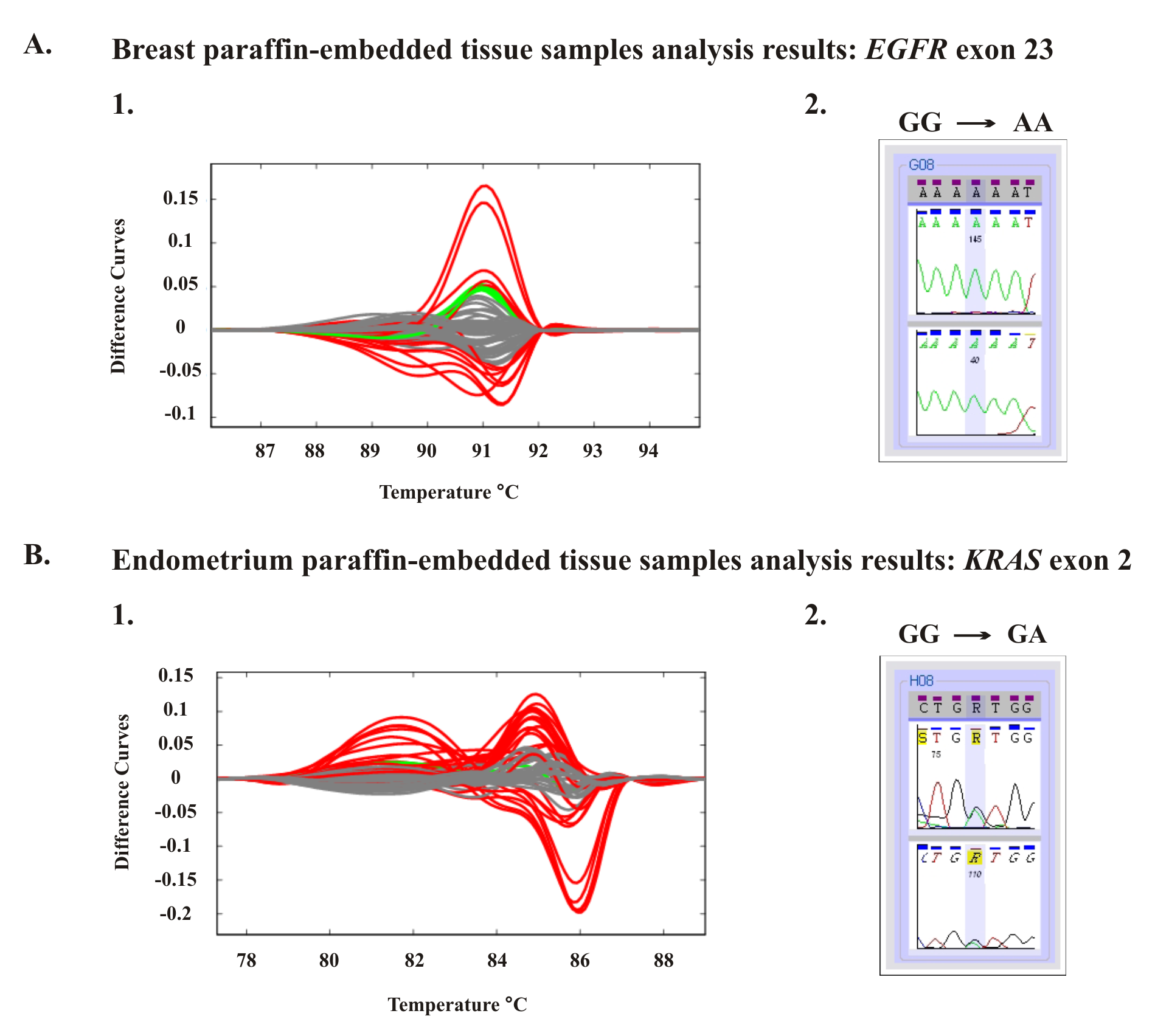

Supplement: Figure S3 — Samples of mutation screening with HRM technology and its validation with sequencing from paraffin-embedded samples. A. One of the assays (EGFR) performed in paraffin-embedded samples from breast cancer: 1. Normalized HRM curves of the assay; 2. Segment of sample assembled trace after sequencing, with the presence of a variant, where AA has replaced both alleles GG. B. Endometrial paraffin-embedded samples for KRAS. 1. Normalized HRM curves of the assay with elevated number of positives samples observed in the HRM curves from paraffin specimens compare to frozen samples; 2. Genotype GG has been substituted by GA. (9.83 MB TIF) [file pone.0014522.s003.tif]
