# Supplementary material for: Detection of Somatic Mutations by High-Resolution DNA Melting (HRM) Analysis in Multiple Cancers
Source: PLoS One. 2011 Jan 17;6(1):e14522. doi: 10.1371/journal.pone.0014522 (PMC3022009; doi:10.1371/journal.pone.0014522)
Supplement: Table S1 — Mutation details from the sequencing analysis of paraffin-embedded samples. (0.09 MB DOC) [file pone.0014522.s004.doc]

| Gene | Exon | Tissue | Location | Nucleotide | Known SNP | Samples affected |
| --- | --- | --- | --- | --- | --- | --- |
| *PIK3CA* | 10 | Breast^1^ | 180418723 | G/A | No | 1 |
|  | 10 | Breast^2^ | 180418764 | G/A | No | 1 |
|  | 10 | Breast  Endometrium | 180418776 | G/A | No | 2  1 |
|  | 10 | Breast^2^ | 180418816 | G/A | No | 1 |
|  | 10 | Breast^1^ | 180418834 | G/A | No | 1 |
|  | 10 | Endometrium | 180418636 | C/T | No | 1 |
| *FGFR3* | 13 | Ovary | 1777612 | G/T | No | 2 |
|  | 13 | Breast | 1777689 | G/T | rs28928868 | 1 |
|  | 13 | Breast  Ovary | 1777720 | G/A | rs3135898 | 1  1 |
| *ERBB2* | 25 | Breast | 35134825 | C/T | No | 1 |
|  | 25 | Endometrium | 35134840 | C/T | No | 1 |
|  | 25 | Breast  Ovary^3^ | 35134869 | C/T | No | 1  1 |
|  | 25 | Breast | 35134902 | C/T | No | 1 |
|  | 25 | Ovary^3^ | 35134939 | C/T | No | 1 |
|  | 25 | Breast | 35134954 | G/A | No | 1 |
|  | 25 | Breast | 35134960 | G/A | No | 1 |
| *TP53* | 5(1) | Ovary | 7519281 | A/G | No | 1 |
|  | 5(1) | Breast^4^ | 7519277 | C/T | No | 1 |
|  | 5(1) | Breast^4^ | 7519262 | C/T | No | 1 |
|  | 5(1) | Breast | 7519249 | C/T | No | 1 |
|  | 5(1) | Breast | 7519206 | C/T | No | 1 |
|  | 5(1) | Ovary | 7519186 | G/T | No | 1 |
|  | 5(1) | Ovary | 7519179 | C/T | No | 1 |
|  | 5(1) | Breast | 7519167 | A/G | TP53-79  (Poly-0007684) | 1 |
|  | 5(2) | Breast | 7519235 | Del(c) | No | 1 |
|  | 5(2) | Breast  Endometrium | 7519131 | G/A | rs28934578 | 1  1 |
|  | 5(2) | Breast | 7519124 | C/T | No | 1 |
| *TP53* | 7 | Breast | 7518284 | C/T | rs28934573 | 1 |
|  | 7 | Breast | 7518259 | Del(g) | rs28934571 | 1 |
| *EGFR* | 23 | Endometrium  Ovary | 55226944 | C/T | rs17290559 | 1  2 |
|  | 23 | Endometrium | 55226980 | G/A | No | 1 |
|  | 23 | Breast | 55227038 | G/A | No | 1 |
|  | 23 | Endometrium | 55227056 | G/A | No | 1 |
|  | 23 | Endometrium | 55227069 | G/A | No | 1 |
| *KRAS* | 2 | Breast | 25289591 | C/T | No | 1 |
|  | 2 | Endometrium | 25289552 | G/T | No | 1 |
|  | 2 | Endometrium  Ovary | 25289551 | G/T/A | No | 10  8 |
|  | 2 | Endometrium  Ovary | 25289548 | G/A | No | 1  1 |
|  | 2 | Breast  Endometrium | 25289546 | G/A | No | 1  1 |
| *BRAF* | 15 | Endometrium | 140099597 | C/T | No | 1 |
| *GATA3* | 5 | Breast | 8151437 | C/T | No | 1 |
|  | 5 | Ovary | 8151426 | C/T | No | 1 |
|  | 5 | Breast | 8151549 | C/T | No | 1 |
| *GATA3* | 6(1) | - | - |  | - | - |
|  | 6(2) | - | - | - | - | - |

^1,2,3,4^ These mutation/variants were found in a single sample.
