# Supplementary material for: Detection of Somatic Mutations by High-Resolution DNA Melting (HRM) Analysis in Multiple Cancers
Source: PLoS One. 2011 Jan 17;6(1):e14522. doi: 10.1371/journal.pone.0014522 (PMC3022009; doi:10.1371/journal.pone.0014522)
Supplement: Table S2 — New nucleotides changes found on the study samples submitted to dbSNP (build 131). (0.09 MB DOC) [file pone.0014522.s005.doc]

| dbSNP(ss) | HGVS nomenclature | Allele | RefSNP(rs) | Gene | Chr | Chromosomal Position Build 131 |
| --- | --- | --- | --- | --- | --- | --- |
| 218178066 | NG_012113.1:g.74632C>T | C/T | rs104886002 | *PIK3CA* | 3 | 178935942 |
| ss218178063 | NG_012113.1:g.74719G>A | A/G | rs104885999 | *PIK3CA* | 3 | 178936029 |
| ss218178085 | NM_006218.2:c.1769G>A | A/G | rs104886021 | *PIK3CA* | 3 | 178936080 |
| p | NG_012113.1:g.74772G>A | A/G | p | *PIK3CA* | 3 | p |
| p | NG_012113.1:g.74772G>A | A/G | p | *PIK3CA* | 3 | p |
| ss218178067 | NG_012113.1:g.74781G>A | A/G | rs104886003 | *PIK3CA* | 3 | 178936091 |
| ss218178064 | NG_012113.1:g.74812G>A | A/G | rs104886000 | *PIK3CA* | 3 | 178936122 |
| ss218178065 | NG_012113.1:g.74830G>A | A/G | rs104886001 | *PIK3CA* | 3 | 178936140 |
| ss218178086 | NM_006218.2:c.1821+51A>T | A/T | rs104886022 | *PIK3CA* | 3 | 178936173 |
| ss218178087 | NM_022965.2:c.1793G>T | G/T | rs104886023 | *FGFR3* | 4 | 1807814 |
| ss218178088 | NM_022965.2:c.1799G>A | A/G | rs104886024 | *FGFR3* | 4 | 1807820 |
| ss218178068 | NG_012632.1:g.17790C>T | C/T | rs104886004 | *FGFR3* | 4 | 1807828 |
| ss218178069 | NG_012632.1:g.17811C>G | C/G | rs104886005 | *FGFR3* | 4 | 1807849 |
| ss218178070 | NG_012632.1:g.17838C>T | C/T | rs104886006 | *FGFR3* | 4 | 1807876 |
| ss218178076 | NG_007726.1:g.177762G>A | A/G | rs104886012 | *EGFR* | 7 | 55259486 |
| ss218178077 | NG_007726.1:g.177820G>A | A/G | rs104886013 | *EGFR* | 7 | 55259544 |
| p | NG_007726.1:g.177851G>A | A/G | p | *EGFR* | 7 | p |
| ss218178090 | NM_005228.3:c.2866G>A | A/G | rs104886026 | *EGFR* | 7 | 55259562 |
| ss218178078 | NG_007726.1:g.177851G>A | A/G | rs104886014 | *EGFR* | 7 | 55259575 |
| ss218178079 | NG_007873.1:g.176437C>T | C/T | rs104886015 | *BRAF* | 7 | 140453128 |
| ss218178080 | NG_015859.1:g.19754C>T | C/T | rs104886016 | *GATA3* | 10 | 8111420 |
| ss218178094 | NM_001002295.1:c.1482-5C>T | C/T | rs104886030 | *GATA3* | 10 | 8111431 |
| ss218178081 | NG_015859.1:g.19877C>T | C/T | rs104886017 | *GATA3* | 10 | 8111543 |
| ss218178093 | NM_033360.2:c.176C>T | C/T | rs104886029 | *KRAS* | 12 | p |
| ss218178092 | NM_033360.2:c.216G>A | A/G | rs104886028 | *KRAS* | 12 | p |
| ss218178091 | NM_033360.2:c.219G>A | A/G | rs104886027 | *KRAS* | 12 | p |
| p | NG_007524.1:g.10570G>T | G/T | p | *KRAS* | 12 | p |
| p | NG_007524.1:g.10570G>A | A/G | p | *KRAS* | 12 | p |
| p | NG_007524.1:g.10570G>T | G/T | p | *KRAS* | 12 | p |
| p | NG_007524.1:g.10570G>A | A/G | p | *KRAS* | 12 | p |
| p | NG_007524.1:g.10576G>A | A/G | p | *KRAS* | 12 | p |
| p | NG_007524.1:g.10576G>A | A/G | p | *KRAS* | 12 | p |
| ss218178071 | NG_007503.1:g.41907C>T | C/T | rs104886007 | *ERBB2* | 17 | 37881299 |
| ss218178072 | NG_007503.1:g.41922C>T | C/T | rs104886008 | *ERBB2* | 17 | 37881314 |
| ss218178075 | NG_007503.1:g.41936G>A | A/G | rs104886011 | *ERBB2* | 17 | 37881328 |
| p | NG_007503.1:g.41951C>T | C/T | p | *ERBB2* | 17 | p |
| p | NG_007503.1:g.41951C>T | C/T | p | *ERBB2* | 17 | p |
| ss218178073 | NG_007503.1:g.41984C>T | C/T | rs104886009 | *ERBB2* | 17 | 37881376 |
| ss218178074 | NG_007503.1:g.42021C>T | C/T | rs104886010 | *ERBB2* | 17 | 37881413 |
| ss218178089 | NM_001005862.1:c.3056G>A | A/G | rs104886025 | *ERBB2* | 17 | 37881434 |
| p | NG_017013.1:g.17331G>T | G/T | p | *TP53* | 17 | p |
| p | NG_017013.1:g.17394G>A | A/G | p | *TP53* | 17 | p |
| p | NG_017013.1:g.17382_17393del12 | del | p | *TP53* | 17 | p |
| p | NG_017013.1:g.17384_17393del10 | del | p | *TP53* | 17 | p |
| p | NG_017013.1:g.17301A>G | A/G | p | *TP53* | 17 | p |
| p | NG_017013.1:g.17305G>T | G/T | p | *TP53* | 17 | p |
| p | NG_017013.1:g.17320C>T | C/T | p | *TP53* | 17 | p |
| p | NG_017013.1:g.17333C>T | C/T | p | *TP53* | 17 | p |
| p | NG_017013.1:g.17376C>T | C/T | p | *TP53* | 17 | p |
| p | NG_017013.1:g.17396C>T | C/T | p | *TP53* | 17 | p |
| p | NG_017013.1:g.17403C>T | C/T | p | *TP53* | 17 | p |
| p | NG_017013.1:g.17347del1 | del | p | *TP53* | 17 | p |
| p | NG_017013.1:g.17458C>T | C/T | p | *TP53* | 17 | p |
| p | NG_017013.1:g.18265C>T | C/T | p | *TP53* | 17 | p |
| p | NG_017013.1:g.18267A>C | A/C | p | *TP53* | 17 | p |
| p | NG_017013.1:g.18348G>A | A/G | p | *TP53* | 17 | p |

p : Pending of validation and integration in dbSNP database, build 131.
